# Improve oxidation resistance at high temperature by nanocrystalline surface layer

**DOI:** 10.1038/srep13027

**Published:** 2015-08-13

**Authors:** Z. X. Xia, C. Zhang, X. F. Huang, W. B. Liu, Z. G. Yang

**Affiliations:** 1 Shagang School of Iron and Steel, Soochow University, Suzhou, 215021, China; 2 School of Materials Science & Engineering, Tsinghua University, Beijing, 100084, China; 3 College of Materials Science and Engineering, Sichuan University, Chengdu, 610065, China

## Abstract

An interesting change of scale sequence occurred during oxidation of nanocrystalline surface layer by means of a surface mechanical attrition treatment. The three-layer oxide structure from the surface towards the matrix is Fe_3_O_4_, spinel FeCr_2_O_4_ and corundum (Fe,Cr)_2_O_3_, which is different from the typical two-layer scale consisted of an Fe_3_O_4_ outer layer and an FeCr_2_O_4_ inner layer in conventional P91 steel. The diffusivity of Cr, Fe and O is enhanced concurrently in the nanocrystalline surface layer, which causes the fast oxidation in the initial oxidation stage. The formation of (Fe,Cr)_2_O_3_ inner layer would inhabit fast diffusion of alloy elements in the nanocrystalline surface layer of P91 steel in the later oxidation stage, and it causes a decrease in the parabolic oxidation rate compared with conventional specimens. This study provides a novel approach to improve the oxidation resistance of heat resistant steel without changing its Cr content.

9% Cr martensitic heat resistant steels such as P91 steel have been considered as the primary candidate structural materials for advanced fossil fired power plants and generation IV nuclear power plants. Due to long-term exposures to high temperatures, the high temperature creep resistance and high temperature oxidation resistance of P91 steel should be simultaneously improved. A Cr content of around 9 wt% in martensitic heat resistant steels is required for the optimization in creep properties, and creep resistances of 9Cr heat resistant steels could be further improved by the control of precipitation behaviors^1^,^2^. It is reported that increasing the Cr content in excess of about 12 wt% is effective in inhibiting the growth of oxide scale for martensitic heat resistant steels^3^. Some researchers even suggest Cr-enriched (Fe,Cr)_2_O_3_ protective scale could form on surface layer of the heat resistant steels with Cr content in excess of 17 wt%^4^,^5^. With increasing Cr content, the oxidation resistance would be improved obviously in heat resistant steels. However, the Cr content in excess of 12 wt% will induce the formation of δ ferrite, which is detrimental to mechanical properties, Therefore, traditional techniques and methods such as improving Cr content hardly resolve the contradictions of the component requirement between creep resistance and oxidation resistance in martensitic heat resistant steels. Grain refinement is an advantageous approach, to increase the oxidation resistance of heat resistant steels in steam without the necessity of increasing the alloy Cr content^6^. Fortunately, if only the microstrcture in the surface layer is refined at the nanometer scale by mean of a surface mechanical attrition treatment (SMAT),which may increase the oxidation resistance of heat resistant steels, the negative effect of the nanocrystalline surface layer on mechanical properties can been neglected^7^.

The nanocrystalline structure with a large number of grain boundaries, which can act as fast atomic diffusion channels^8^,^9^. Compared to diffusions in materials with conventional grain sizes, greatly enhanced atomic diffusivities have been reported in nanocrystalline materials^10^,^11^. Hence, it is expected that SMAT, which can produce nanocrystalline layer on the surface of steels, can significantly improve oxdiation resistance. In the present work, the high temperature oxidation behavior of nanocrystallined P91 steal is studied, and effect of nano-grain boundary on the formation of oxide scale is illustrated, all of which can provide theoretical supports for the improvement of oxidation behavior in heat resistant steels exposed to high-temperature and high-pressure water vapor.

The chemical composition of commercial P91 steel used in the present work is 0.1 C-0.6 Mn-8.8 Cr-0.95 Mo. A polished plate specimen (Ф50 mm ×4.0 mm in size) of a tempered P91 steel with conventional grain size of 14 μm was subjected to SMAT. SMAT was performed only on one side of the specimens. The set-up and procedure were described as follows: hardened 0.8 C steel balls, with 5 mm in diameter and mirror-like surfaces, were placed in a reflecting chamber that was vibrated by a vibration generator with a frequency of 20 kHz. After being treated for 30 min, the surface roughness of SMAT-ed sample is comparable to that of the original, unpolished specimen. Other details of the experimental setup and the SMAT processing are described in the reference^12^.

In order to make comparisons of the oxidation resistance between convertional materials and SMAT-ed nanocrystalline materials, oxidation specimens with 10 × 20 × 2 mm^3^ were cut by an electrical discharge machine for the nanocrystalline specimens and conventional grain specimens. The surfaces of specimens, except for the SMAT treated surface, were polished to mirror-like surface by 2.5 μm Al_2_O_3_ polishing paste, then cleaned in distilled water and then in acetone with ultrasonic agitation for 15 min prior to oxidation. Oxidation behaviors were tested in supercritical water oxidation equipments operated at 848 K and 14.1 MPa, and the total time of oxidation was up to 48 days (1152 h). Each oxidation date at different time was calculated by averaging the value from three specimens. Oxide scale were characterized by SEM, EPMA, XRD and TEM. Cross-sectional morphologies of the specimens were observed by using SEM with back-scattered electron (BSE) and Electron probe micro-analyzer (EPMA). Microstructure characterizations of nanocrystalline microstructure were also examined using TEM. Thin foil specimens for TEM observation were prepared using Focused Ion Beam instrument (FIB).

It is reported that the grains in the surface layer region within 100 μm thick are refined into nanometer scale, and the thermal stability of nanocrystalline microstructure is excellent until the temperature above 1033 K^7^. The time dependence of weight gain was plotted as a function of time in Fig. 1. The weight gain data of the conventional specimens were fitted to the parabola law relation given in Eq. (1):


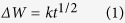


Where *W* is the weight gain in mg/cm^2^, *k* is the oxidation rate constant in mg/cm^2^/h, and *t* is time in hour. The oxidation rate constant *k* is 0.044 for the conventional specimens. However, the weight gain data (24 h) of the nanocrystalline specimens could not fitted to the parabola or a power law in Fig. 1a. Because the diffusivity of Cr in the nanocrystal grain is 4–5 orders of magnitude higher than that in the conventional grain^12^ , both the diffusivity of oxygen and iron sharply increase in the nanocrystalline P91 steel. Therefore, P91 steel with nanocrystalline microstructure rapidly forms a double layer of Fe_3_O_4_ and FeCr_2_O_4_, and this will lead to an acceleration of the oxidation rate in the early stage before the formation of a more protective Cr-rich (Fe, Cr)_2_O_3_ oxide scale as shown in Fig. 2a. The data analysis of weight gain was considered as a starting from the 24 h oxidation specimen, the oxidation kinetics of the nanocrystalline exhibited parabolic law after the formation of the (Fe, Cr)_2_O_3_ inner layer on the nanocrystalline surface layer of P91 steel as shown in Fig. 2b–d, which causes a decreasing in the oxidation rate constant *k* from 0.044 in the conversional specimen to 0.026 in the nanocrystalline specimen (Fig. 1b), and this denotes a notable improvement of oxidation resistance on the nanocrystalline surface layer.

To verify the results on the formation of (Fe, Cr)_2_O_3_, the chemical composition profiles of the oxide scale on both the conventional and the nanocrystalline specimens were examined using EPMA map/line-scan technique, as shown in Fig. 3. The oxide layer needs to be thick enough for EPMA analysis, so specimens exposed after 1152 h were selected. The three-layer oxide structure from the surface towards the alloy consisted of an Fe_3_O_4_ outer layer, a spinel FeCr_2_O_4_ in the middle, and a corundum (Fe, Cr)_2_O_3_ inner layer at the oxide/alloy interface. This is different from the typical two-layer scale consisting of an Fe_3_O_4_ outer layer and an FeCr_2_O_4_ inner layer on the conventional surface of P91 steel^13^,^14^. The O, Cr and Fe concentrations in the Fe_3_O_4_ and FeCr_2_O_4_ are similar in both the conventional and nanocrystalline specimens. However, the inner oxide scale (Cr, Fe)_2_O_3_ is richer in Cr, which can be clearly seen from the cross-sectional scale morphology in Fig. 3b. The XRD profiles of the conventional and nanocrystalline specimens exposed after 576 h are shown in Fig. 4a,b, respectively. Fe_3_O_4_, FeCr_2_O_4_ and (Fe, Cr)_2_O_3_ were all detected. Further analysis was performed using TEM to verify the morphology and phase structure of different types of oxides. The nanocrystalline specimen exposed after 576 h was selected to prepare the TEM foils because of the processing depth limitation in the FIB instrument. The selected area electron diffraction pattern (SAED) of TEM indicated that the oxide contained the spinel FeCr_2_O_4_ and the corundum (Fe, Cr)_2_O_3_ in Fig. 4c. The Cr concentration increased from 14.5 wt% in FeCr_2_O_4_ to 31.4 wt% in (Fe, Cr)_2_O_3_. The map-scan profiles also proved that the inner oxide scale (Fe, Cr)_2_O_3_ is richer in Cr (Fig. 4d–g).

Cr diffusion in the metal matrix and oxide is not fast enough for a stable corundum (Fe, Cr)_2_O_3_ scale in 9 Cr steel to form at 848 K. The scales of 10 Cr steel typically consist of an outer Fe_3_O_4_ layer and an inner layer containing (Fe, Cr)_3_O_4_ at 923 K in Ar + 50%H_2_O^15^. A scale composed of Fe_2_O_3_, Fe_3_O_4_, and (Fe, Cr)_3_O_4_ is formed on P91 steel at 923 K in steam atmospheres, and it is still composed of Fe_3_O_4_ and (Fe, Cr)_3_O_4_ at 1073 K^16^. These reports are in good agreement with the results of the conventional P91 steel specimens in this work. Although, the tendency for Cr to be selectively oxidized becomes more pronounced as diffusion in the alloy becomes enhanced with increasing temperature, the Cr concentration in 9 Cr steel is still not sufficiently high to allow the formation of a more protective (Fe, Cr)_2_O_3_ layer. However, the scale is composed mainly of (Fe, Cr)_2_O_3_ on AISI 430 steel with 16.21 wt% Cr content at 923 K, and a layer of Cr_2_O_3_ is formed at 1073 K owing to the higher diffusion rate of Cr at this temperature than at 923 K^16^. In this work, A protective (Fe, Cr)_2_O_3_ layer could form on the nanocrystalline surface layer in P91 steel with 8.8 wt% Cr at 848 K.

The oxidation process of the nanocrystalline surface layer is divided into two stages, namely before and after the formation of a protective (Fe, Cr)_2_O_3_ layer. Before the formation of the (Fe, Cr)_2_O_3_ layer, the diffusivity of oxygen and iron sharply increase on the nanocrystalline surface layer of P91 steel. So oxidation rates of the nanocrystalline specimens are significantly accelerated in the initial stage. Increase of the oxidation rate with decreasing grain size is attributed to the larger grain-boundary area, which result in an increase in the short circuit diffusion paths^17^.The similar results showed that SMAT had a negative effect on the corrosion resistance of Fe^18^. In contrast, after the formation of a protective (Fe, Cr)_2_O_3_ layer, due to its higher density of grain boundaries in the nanocrystalline surface layer, a higher flux of Cr goes towards the (Fe, Cr)_2_O_3_-the alloy interface, while the spinel (Fe, Cr)_2_O_3_ and FeCr_2_O_4_ layer inhabits Cr and O/Fe diffusion through it. Therefore, the (Fe, Cr)_2_O_3_ scale at the alloy-oxide interface has a higher concentration of Cr. O and Fe diffusivity would also decrease due to the formation of (Fe,Cr)_2_O_3_ inner layer in the nanocrystalline surface layer of P91 steel. The enhancement of Cr diffusion guarantee the stable growth of the Cr-rich (Fe, Cr)_2_O_3_ scale, which significantly improve oxidation resistance. Even a Cr_2_O_3_ scale may be formed at higher temperatures owing to the higher diffusion rate of Cr.

## Additional Information

**How to cite this article**: Xia, Z. X. *et al.* Improve oxidation resistance at high temperature by nanocrystalline surface layer. *Sci. Rep.* **5**, 13027; doi: 10.1038/srep13027 (2015).

## Figures and Tables

**Figure 1:**
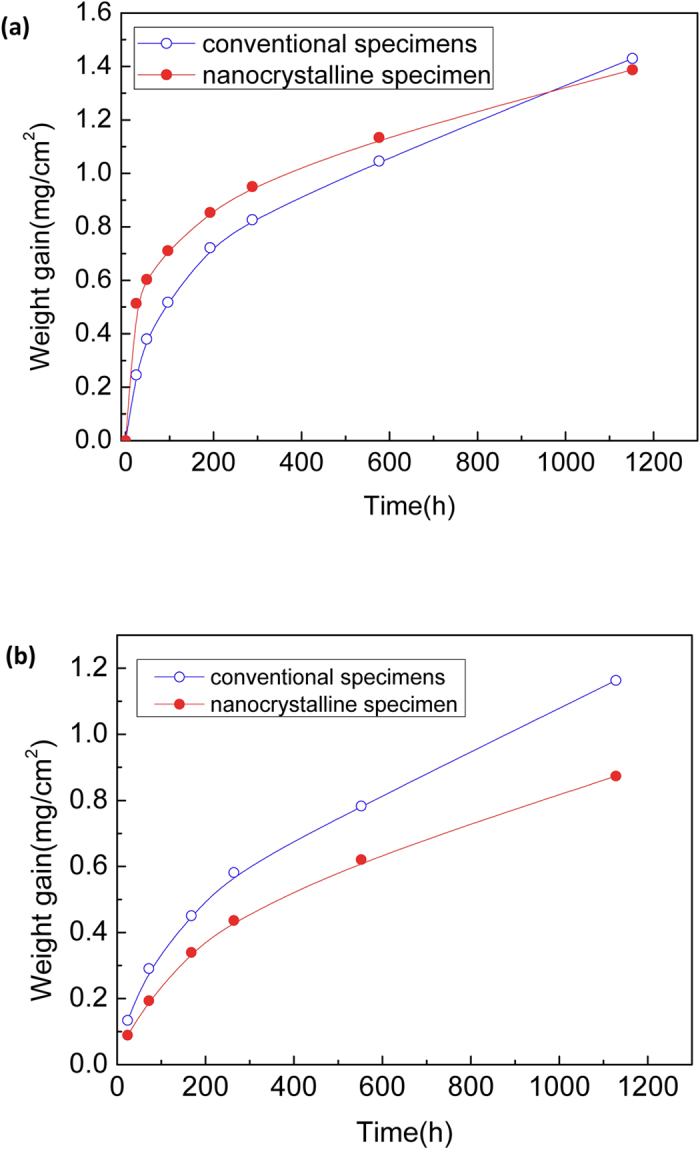
Oxidation kinetics of nanocrystalline and conventional specimens in water vapor with 848 K and 14.1 MPa. The parabola oxidation rate constant *k* is 0.044 in conventional specimens. However, the weight gain data (24 h) of the nanocrystalline specimens is not fitted to a parabola or a power law (**a**). The oxidation kinetics of the nanocrystalline exhibits parabolic law and oxidation rate constant *k* decreases to 0.026 after the data of 24 h oxidation is subtracted using the method of background subtraction (**b**).

**Figure 2:**
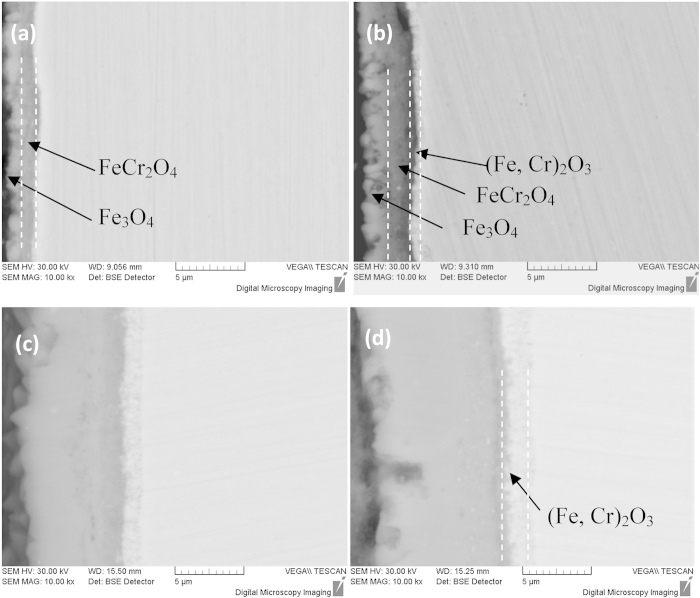
Cross-section back-scattered electron images of the oxide scale formed on the nanocrystalline surface layer exposure at 848 K. (**a**) after 24 h, (**b**) after 96 h, (**c**) after 576 h, (**d**) after 1152 h. The three-layer oxide structure from surface towards matrix is Fe_3_O_4_, spinel FeCr_2_O_4_ and corundum (Fe,Cr)_2_O_3_, The continuous (Fe,Cr)_2_O_3_ inner layer forms on the nanocrystalline surface layer of P91 steel at 848 K for 24 h.

**Figure 3:**
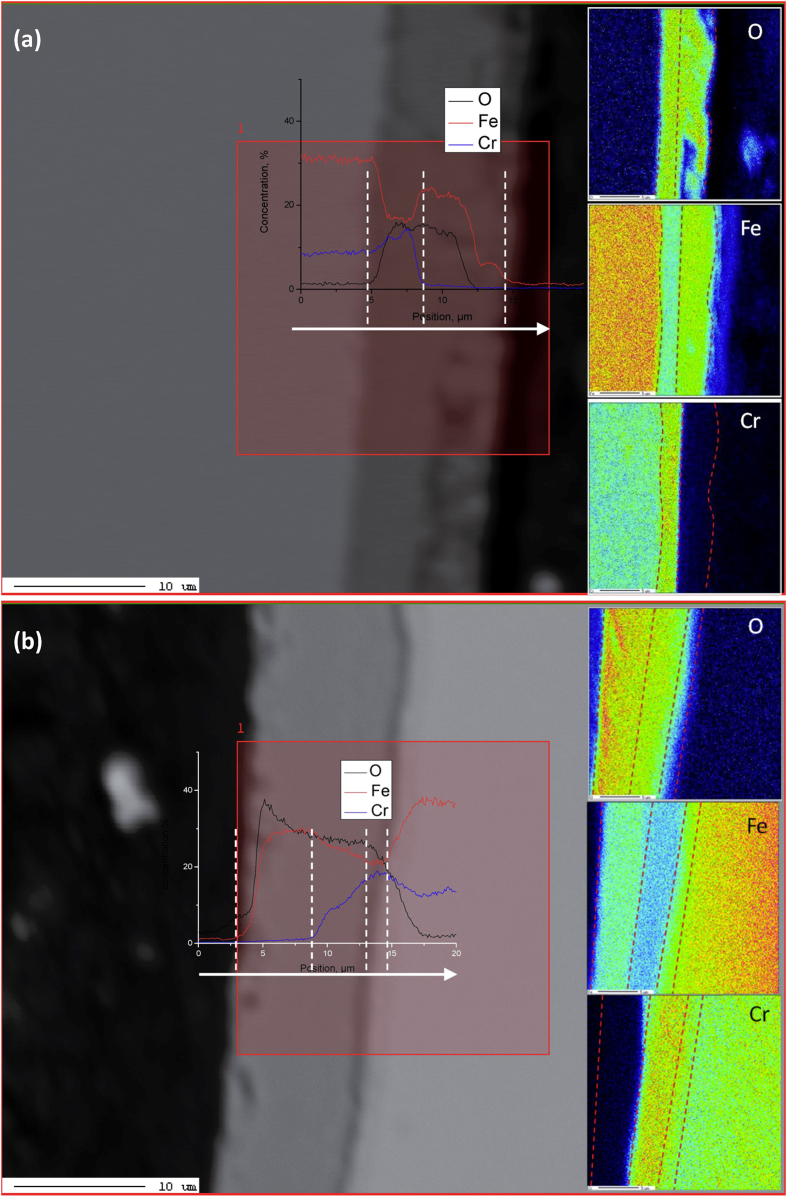
Chemical composition profiles of oxide scale formed on both the conventional (**a**) and nanocrystalline (**b**) specimens surface layer exposure at 848 K for 1152 h are performed using EPMA map/line-scan technique. The three-layer oxide structure from surface towards matrix was Fe_3_O_4_, spinel FeCr_2_O_4_ and corundum (Fe,Cr)_2_O_3_ formed on the nanocrystalline surface layer (**b**), it is different from the typical two-layer scale consisted of Fe_3_O_4_ outer layer and FeCr_2_O_4_ inner layer in conventional P91 steel (**a**). The inner oxide scale (Cr, Fe)_2_O_3_ is rich in Cr, which can be clearly seen from the cross-sectional scale morphology of the nanocrystalline surface layer.

**Figure 4:**
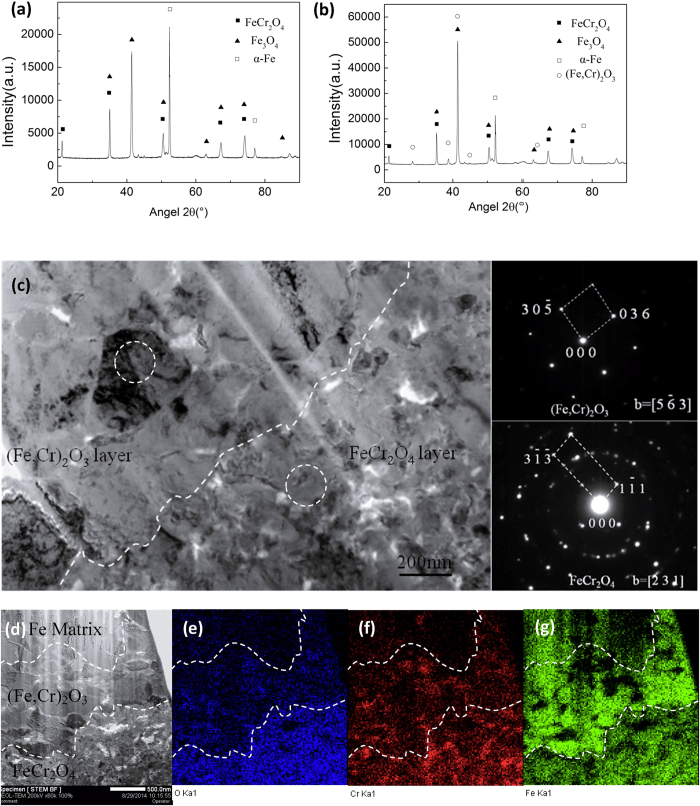
XRD patterns and TEM observation of the oxide scale formed on the nanocrystalline surface layer exposure at 848 K for 576 h. Fe_3_O_4_, FeCr_2_O_4_ were detected in the conventional specimen by XRD (**a**), Fe_3_O_4_, FeCr_2_O_4_ and (Cr, Fe)_2_O_3_.were detected in the nanocrystalline specimen by XRD (**b**). A bright-field TEM image and the electron diffraction pattern show the middle oxide scale and the inner scale are identified as FeCr_2_O_4_ and (Fe,Cr)_2_O_3_, respectively (**c**). The map-scan profiles also prove that the inner oxide scale (Cr, Fe)_2_O_3_ is rich in Cr (**d–g**).
